# Controlled switching of single-molecule junctions by mechanical motion of a phenyl ring

**DOI:** 10.3762/bjnano.6.213

**Published:** 2015-10-30

**Authors:** Yuya Kitaguchi, Satoru Habuka, Hiroshi Okuyama, Shinichiro Hatta, Tetsuya Aruga, Thomas Frederiksen, Magnus Paulsson, Hiromu Ueba

**Affiliations:** 1 Department of Chemistry, Graduate School of Science, Kyoto University, 606-8502 Kyoto, Japan; 2 Donostia International Physics Center (DIPC), 20018 San Sebastián, Spain; 3 IKERBASQUE, Basque Foundation for Science, 48013 Bilbao, Spain; 4 School of Computer Science, Physics and Mathematics, Linnaeus University, 391 82 Kalmar, Sweden; 5 Division of Nano and New Functional Materials Science, Graduate School of Science and Engineering, University of Toyama, 930-8555 Toyama, Japan

**Keywords:** density functional theory, phenyl rings, quantum transport simulations, scanning tunneling microscopy, single-molecule switches

## Abstract

Mechanical methods for single-molecule control have potential for wide application in nanodevices and machines. Here we demonstrate the operation of a single-molecule switch made functional by the motion of a phenyl ring, analogous to the lever in a conventional toggle switch. The switch can be actuated by dual triggers, either by a voltage pulse or by displacement of the electrode, and electronic manipulation of the ring by chemical substitution enables rational control of the on-state conductance. Owing to its simple mechanics, structural robustness, and chemical accessibility, we propose that phenyl rings are promising components in mechanical molecular devices.

## Introduction

Atomic-scale switches are key device components in future electronics for logic gates and memory elements operating in the limit of electronics miniaturization [1–5]. The mechanical control of single atoms has been employed to demonstrate the ultimate limit of electrical switches [6–8], but the use of single molecules as active units would be particularly appealing if device properties (such as switching power, speed, and stability) could be engineered and controlled by chemical design. By optimizing the on-state conductance, a large on/off ratio can be achieved, which is essential for the reliable operation of a molecular switch. In this context, π-conjugated species are promising because π orbitals can be manipulated by introducing functional groups, which illustrates a way to control the junction conductance [9] (e.g., via quantum interference effects [10–11]).

In a previous study [12], using a scanning tunneling microscope (STM), we demonstrated a molecular switch made functional by the mechanical motion of a phenyl ring, which is the atomic-scale analogue of the conventional toggle switch. A phenoxy (C_6_H_5_O, PhO) molecule bonded to the Cu(110) surface was reversibly lifted (released) to (from) the STM tip while being anchored to the surface via an oxygen atom. The reversible switching of the junction allowed us to explore the effect of molecular interaction on the molecular conductance. Here we extend this study and show that switching can be controlled by voltage pulses as well as by mechanical manipulation of the tip. Furthermore, the electronic levels are tunable through chemical manipulation of the phenyl ring, which in turn allows us to tailor the on-state conductance.

## Methods

As described in the previous study [12], the experiments were carried out in an ultrahigh vacuum chamber equipped with an STM operating at 4.5 K. The Cu(110) surface was cleaned by repeated cycles of argon ion sputtering and annealing. PhO and thiophenoxy (C_6_H_5_S, PhS) were prepared on Cu(110) as reported previously [13]. Methyl-substituted phenol compounds (*meta*-cresol and 3,5-xylenol) were exposed to the surface at 300 K, which caused partial dehydrogenation as in the case of phenol. An electrochemically etched tungsten tip was used as an STM probe. The tips were repeatedly and gently touched to the Cu surface to coat them with copper, resulting in Cu-terminated tips for reliable switching [14]. We observed that sharp tips give high-contrast images and can successfully lift up the molecules.

The computational approach was detailed in [12]. Briefly, we used Kohn–Sham density functional theory (DFT) implemented in VASP [15–16] to obtain the atomic structure and total energy using the optPBE-vdW [17] exchange-correlation functional. Electron transport was computed with the DFT-based codes TranSIESTA [18–19] and Inelastica [20] using GGA-PBE [21] for the junctions connected to semi-infinite electrodes. The voltage-induced atomic forces were computed as the difference in the Hellman–Feynman force on each atom between the finite sample voltage, *V*_S_ = ±0.5 V, and zero voltage [19,22]. We define the lateral position of the tip, *x*, as the nominal distance (i.e., before relaxations) along [001] between the Cu tip apex atom and the Cu bridge site containing the chalcogen bond. Also, we define the tip height, *h*, as the distance between the planes containing the third Cu layers of the surface and of the tip pyramid.

## Results and Discussion

**Figure 1:**
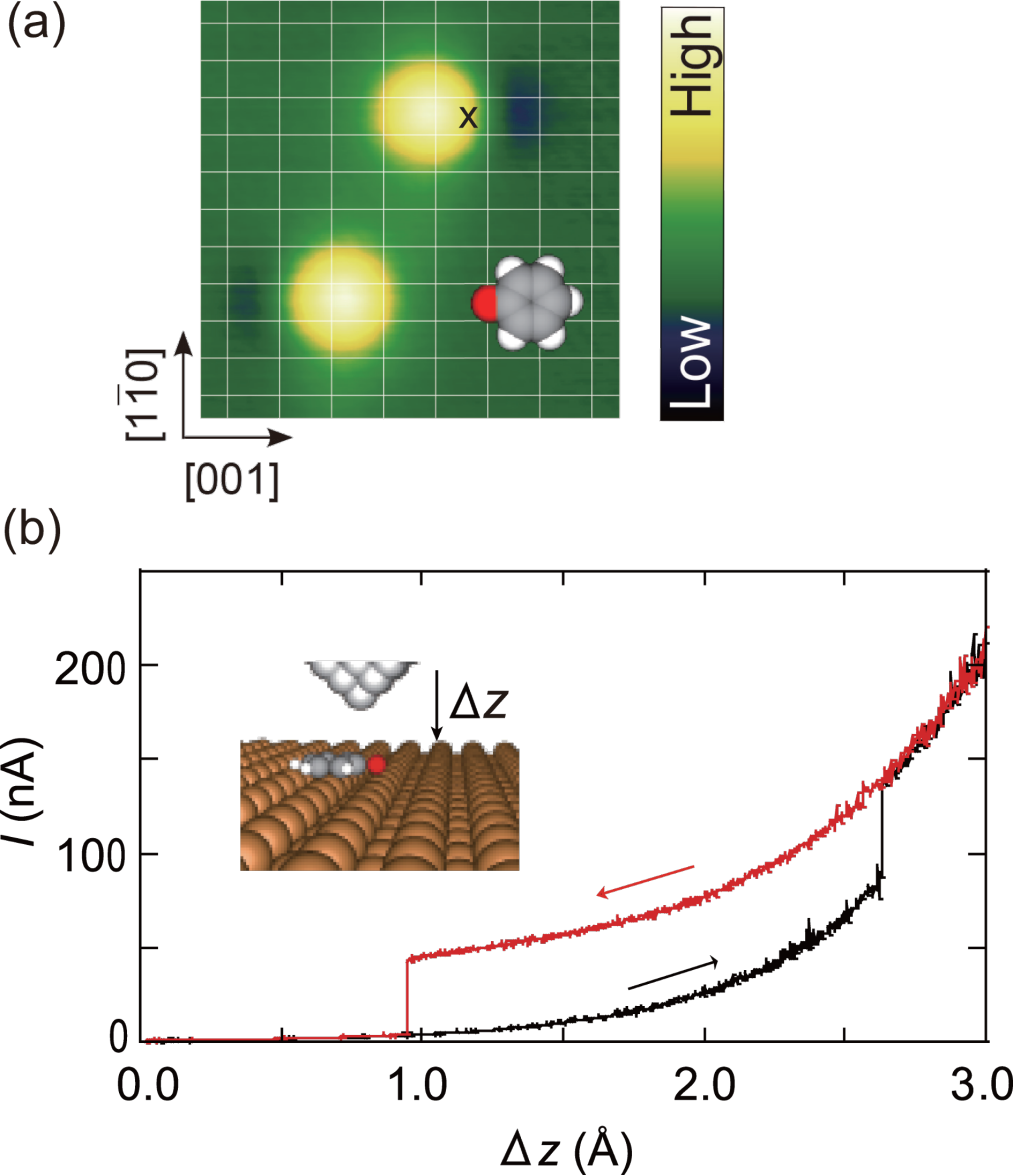
(a) An STM image of PhO molecules on Cu(110) with a schematic illustration superimposed. A protrusion and depression pair associated with the phenyl ring and oxygen atom, respectively. The white grid lines indicate the lattice of Cu(110). The image was obtained at *V*_S_ = 50 mV and *I* = 1 nA (28 × 28 Å^2^). (b) The tunnel current during the approach (black) and retraction (red) of the tip along the surface normal. The tip was first positioned over the protrusion of the top molecule in (a) at a height corresponding to *V*_S_ = 50 mV and *I* = 1 nA, and the feedback loop was turned off. Then the tip was laterally displaced in the [001] direction by 2 Å (indicated by the cross) and moved toward the molecule. The origin of the abscissa (Δ*z* = 0) is the initial tip height and the increase in Δ*z* corresponds to the approach to the molecule, as shown in the inset. Data adapted from [12].

Figure 1a shows the STM images of two PhO molecules on Cu(110) [13]. The images show a depression, which is ascribed to the oxygen atom. The molecules are nearly flat lying and oriented along the [001] direction, as depicted by the illustration. As reported in the previous study [12], the molecule can be lifted up to the tip and reversibly released by controlling the tip–surface distance. The tip was first precisely positioned over the protrusion of the top molecule in Figure 1a at the set point corresponding to *I* = 1 nA at *V*_S_ = 50 mV. After the feedback was turned off, the tip was laterally displaced along the [001] direction by 2 Å (cross over the molecule) and then moved toward the molecule. Figure 1b shows a typical tunnel current recorded during the approach (black) and subsequent retraction (red), where remarkable hysteresis appears when the molecule is successfully lifted and released. Note that Δ*z* is defined as the distance toward the molecule with respect to the initial set point, as depicted in the inset. The current during the approach shows a jump at Δ*z* = 2.6 Å to the high-current state, which returns to the original state at Δ*z* = 1.0 Å during the retraction. The high-current state is ascribed to the lift-up of PhO and the contact formation between tip and molecule [23–27]. After the complete retraction of the tip, we confirm that the molecule returns to its original position by imaging the area. Also we can repeat the lift and release without changing the condition of the tip apex. Thus, the molecular junction can be reversibly switched by mechanical motion of a phenyl ring. As shown in [12] this switching mechanism is also supported by the calculated potential energy landscape as a function of tip approach.

**Figure 2:**
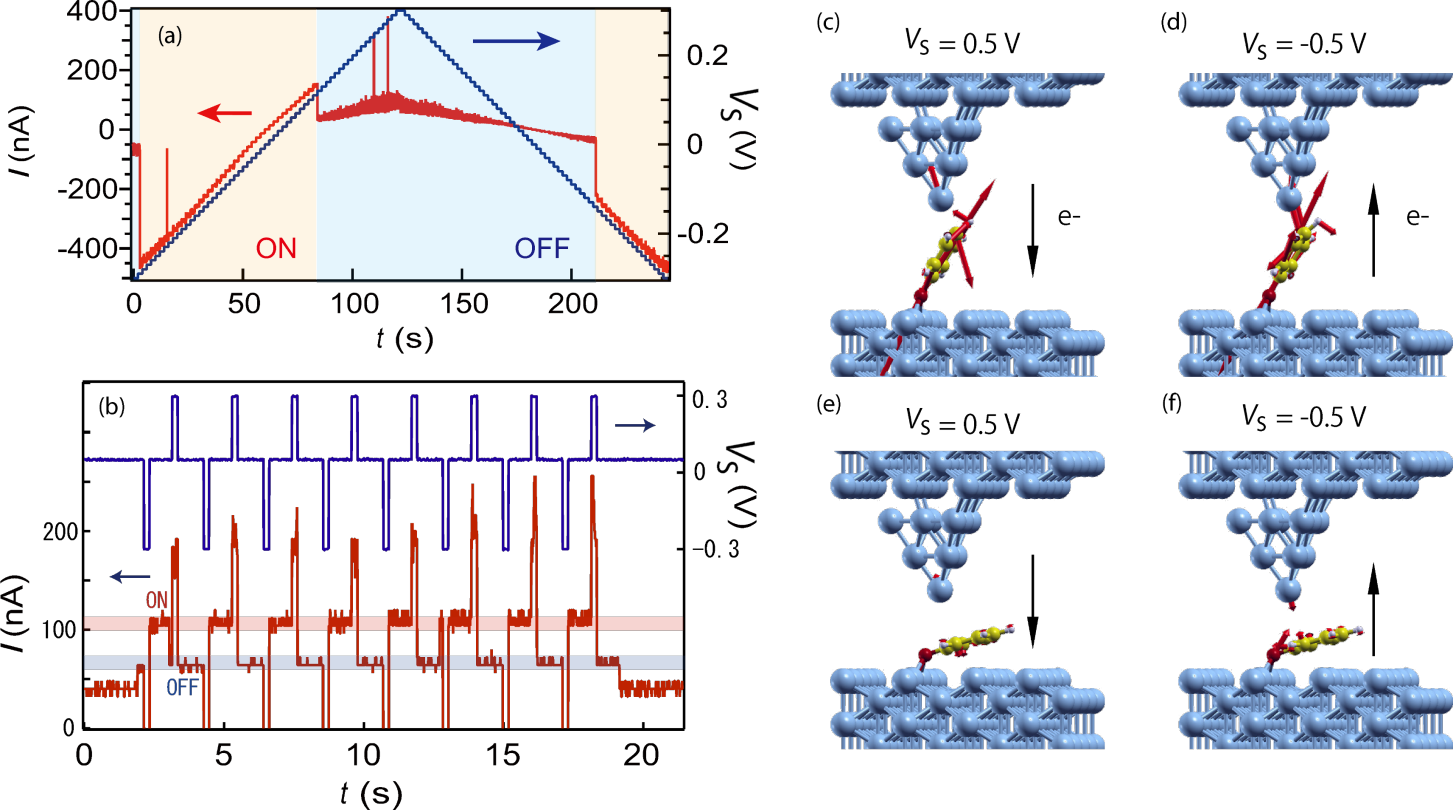
(a) The control of the switch by application of a bias voltage for PhO. The sample voltage *V*_S_ was ramped (blue lines) with the tip fixed over the molecule, during which the tunnel current was recorded (red lines). The on-state (lifted) is favorable at a negative sample bias, whereas the off-state (flat) is favorable at a positive bias. (b) Repeated switching of the molecular junction by using a sequence of voltage pulses. The molecule lifts up and lies down when negative and positive voltages are applied to the substrate (Cu surface), respectively. The atomic forces acting on the molecule in the junction were calculated for the lift-up configuration (*x* = 1.4 Å; *h* = 13.9 Å) with (c) positive and (d) negative sample voltages. Likewise the atomic forces for the lying-down configuration (*x* = 1.4 Å; *h* = 11.9 Å) were calculated with (e) positive and (f) negative sample voltage. The black arrows indicate the direction of the electron flow.

The switch can also be actuated by a bias voltage with the tip position fixed (Figure 2). The sample bias was increased from −0.3 to 0.3 V and subsequently decreased to −0.3 V (blue lines in Figure 2a) while the tip was fixed over the molecule. The tunnel current during the voltage ramp (red lines) shows voltage-dependent two-state switching. The junction was in the off-state at first (*t* = 0, *V*_S_ = −0.3 mV), but immediately switched to the on-state. It was maintained until the voltage reached 0.1 V (*t* = 80 s), where the junction returned to the off-state. The off-state dominates until the voltage decreases to −0.1 V (*t* = 210 s), where the switch was again turned on. As a whole, the junction prefers the on and off states at negative and positive voltage, respectively. Using this voltage-dependent preference, we tried to toggle the switch by applying positive/negative pulse voltages with the electrode distance fixed (Figure 2b). By applying a short pulse of positive voltage to the on-state junction, the molecule switched to the off-state. Conversely, switching to the on-state was achieved with a negative voltage pulse. This switching mechanism is particularly important for possible applications because no complete STM setup to control the tip height is needed.

The bias-dependent behavior was reproduced by our calculations of the induced atomic forces by applying a voltage across the junction (Figure 2c–f). These nonequilibrium forces are evaluated using the nonequilibrium density matrix and Hamiltonian in the equations for the equilibrium forces [19,22]. One observes repulsive (attractive) forces between the tip apex and nearest C-atom at positive (negative) sample voltages, consistent with the electrostatic force to be expected in the applied electric field with an overall negative charge associated with the C-atom. To further investigate this, we computed the following Hirshfeld (Voronoi) atomic charges [28] for the lifted PhO (*x* = 1.4 Å and *h* = 13.9 Å) in equilibrium *V*_S_ = 0: −0.043 (−0.038) for C, 0.032 (0.042) for H, −0.234 (−0.274) for O, and 0.105 (0.073) for the Cu apex atom (in units of *|e|*). These atomic charges were further found not to significantly change with applied voltage *V*_S_ = ±0.5 V. We thus conclude that the direction of the nonequilibrium force can be rationalized by electrostatics.

**Figure 3:**
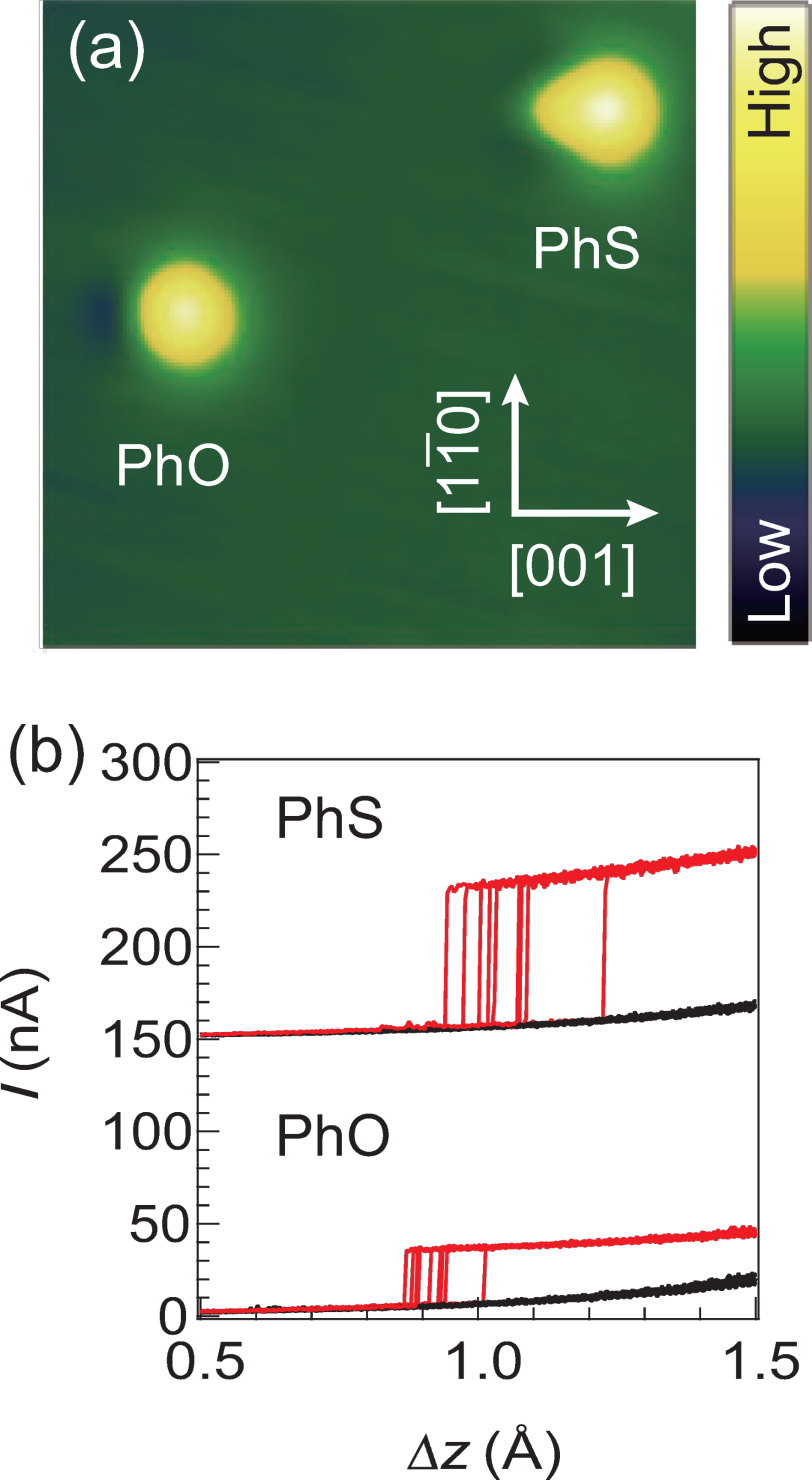
(a) STM image of coadsorbed PhO (left) and PhS (right) obtained at *V*_S_ = 50 mV and *I* = 1 nA (52 × 52 Å^2^). (b) Measured *I*–Δ*z* curves for PhO and PhS (offset). The switching was repeated for 10 cycles for each molecule with the identical tip apex. The height of the plateau (current jump at the cleavage) is defined as the on-state conductance of the molecular switches.

The current plateau that appears just before cleavage of the junction by tip retraction (Figure 1b) provides a well-defined measure of the direct current through the molecule. To investigate the influence of the anchoring group on the conductance, we also prepared PhS on the surface. This molecule is imaged as a protrusion with a tail which is ascribed to the sulfur atom (Figure 3a). The local structure is similar with that of PhO with the phenyl ring oriented along [001] and the sulfur atom bonded to the short-bridge site [13]. The controlled switching of the molecular junction is feasible with PhS as well as PhO, and the conductance values for different molecules are compared with the same tip apex (Figure 3b). As mentioned above, the initial tip height (Δ*z* = 0) was defined by the set point of 50 mV and 1 nA over the protrusion of a PhO molecule. The difference in the set point for different species is calibrated by their topographic height. Thus, the tip–electrode distance is the same between two different junctions at the same Δ*z*. The junction switching was conducted for PhO and PhS molecules placed near each other (Figure 3a) with the same tip apex. The measurement was repeated ten cycles for each molecule, and the current jumps during the retraction were recorded. The height of the plateau, corresponding to the conductance for each molecule, is robust between repeated measurements and is larger by a factor of ≈2 for PhS. This demonstrates that the conductance is modified by chemical substitution of the anchoring atom from oxygen to sulfur.

**Figure 4:**
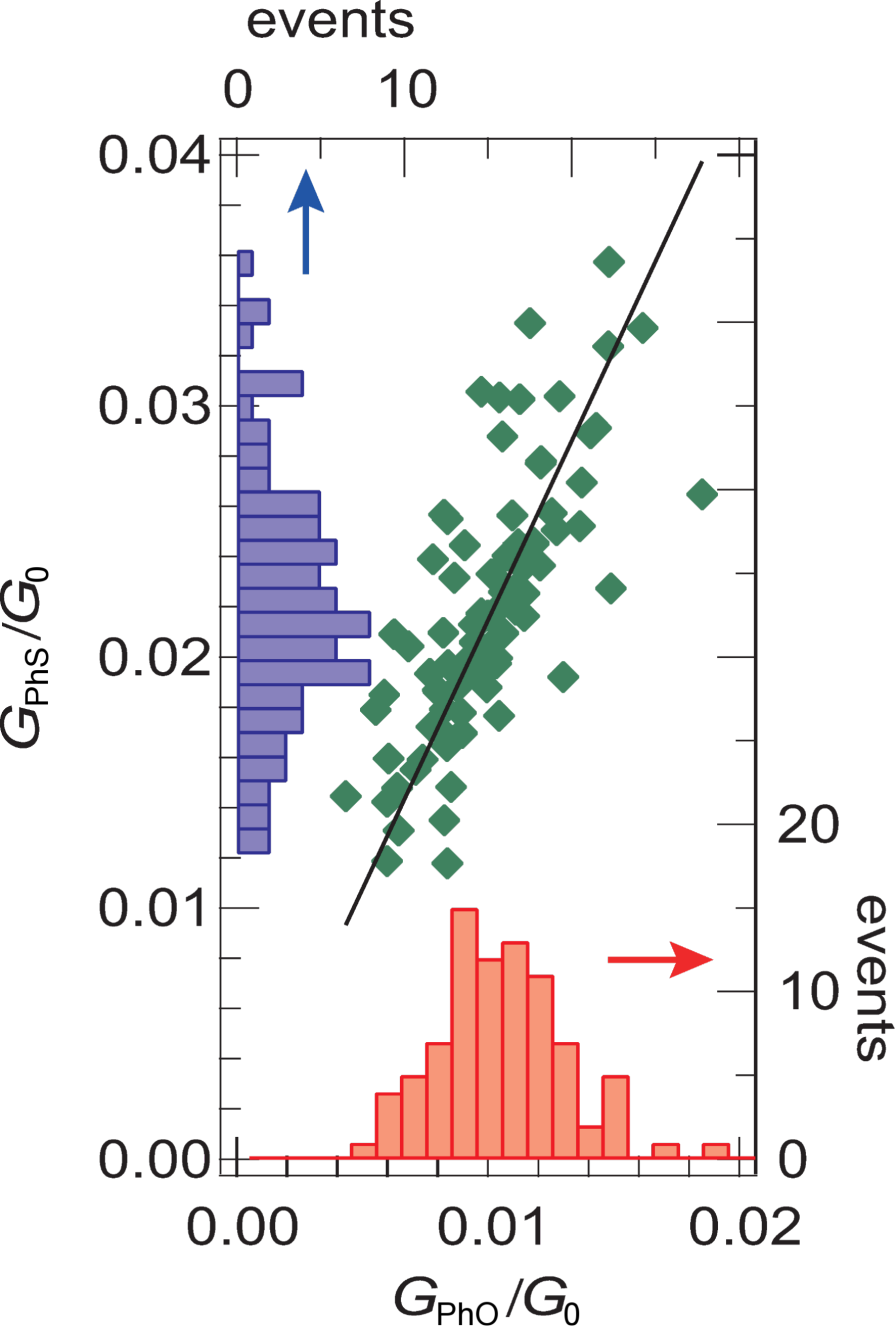
The dependence of the tip–molecule contact on the conductance. For a given tip apex, the conductance is recorded sequentially for each of the two molecules, generating a data point (green diamond) in the plot. Then the tip apex is modified by a gentle touch to the surface and the measurement procedure is repeated. The 85 different tip apexes were employed. Thus, the distribution of the conductance results from the variation of the tip–molecule contact. The solid line shows a linear fit that reveals *G*_PhS_ = 2.1·*G*_PhO_, suggesting that the PhS molecule is more conductive than the PhO molecule by a factor of ≈2.1, irrespective of the tip–molecule contact geometry.

To investigate the dependence of tip–molecule contact geometry on the molecular conductance, we obtained data for PhO and PhS with modified tip apexes. First, we recorded the conductance for each of the two molecules before the tip apex was modified by a gentle touch to the surface to change the tip–molecule contact geometry. We performed such conductance measurements with 85 modified tip apexes and obtained the distribution shown in Figure 4. The conductance values, which depend on the tip apex, are distributed at (1.0 ± 0.3) × 10^−2^*G*_0_ and (2.2 ± 0.5) × 10^−2^*G*_0_, for PhO and PhS molecules, respectively, where *G*_0_ is the quantum of conductance. The variation of the conductance results from the difference in the tip–molecule contact geometry. Nevertheless, the linear relation in Figure 4 indicates that PhS is more conductive than PhO by a factor of 2.1, irrespective of the details of the tip–molecule contact.

**Figure 5:**
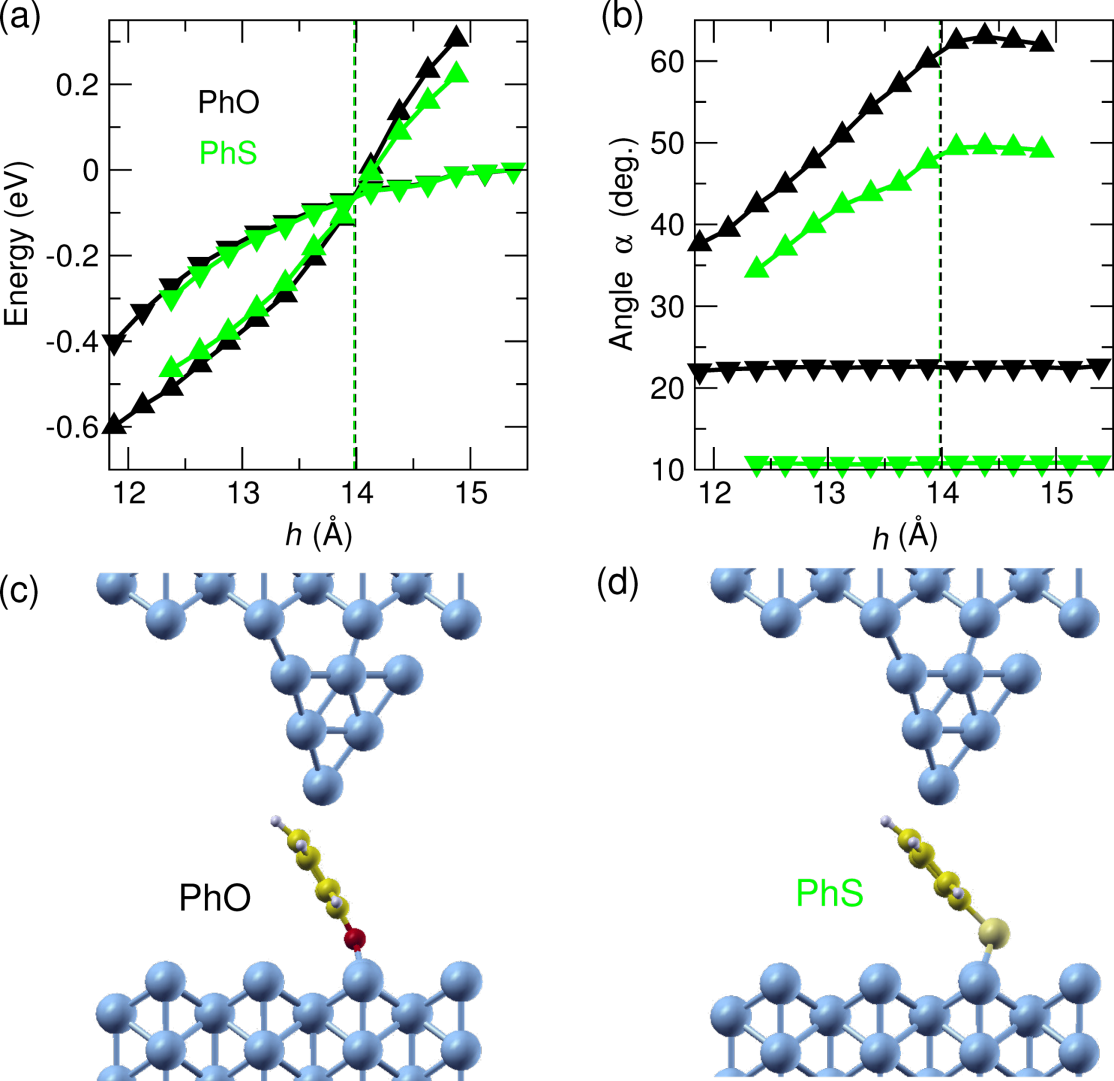
Stability diagram and structure for PhO and PhS switching. (a) Total energy difference and (b) molecular tilt angle with the optPBE-vdW functional as a function of tip height with the lateral position fixed at *x* = 1.4 Å (black: PhO adapted from [12]; green: PhS; down-facing triangles: molecule flat, upright triangles: molecule lifted up). Vertical dashed lines indicate the tip height where flat and lifted configurations are energetically equal. (c) PhO (adapted from [12]) and (d) PhS molecules in the lifted configuration (*h* = 13.9 Å).

The role of the anchoring group (O vs S) on the junction geometry was also computed. Figure 5 compares the total energy differences and molecular tilt angles for PhO [12] and PhS junctions in flat and tilted configurations. While the energy difference is rather similar, the tilt angle is generally lower for PhS than for PhO for the same tip height. This difference is due to different binding patterns to the Cu(110) surface (Figure 5c,d). For PhS the sp^3^ hybridization of the S-atom results in a rather perfect tetrahedral bonding motif with the Cu–S–C bond angle being 111° (*h* = 13.9 Å). In the case of PhO, the sp^3^ hybridization of the O-atom is more distorted and the corresponding Cu–O–C bond angle is found to be 135° (*h* = 13.9 Å).

**Figure 6:**
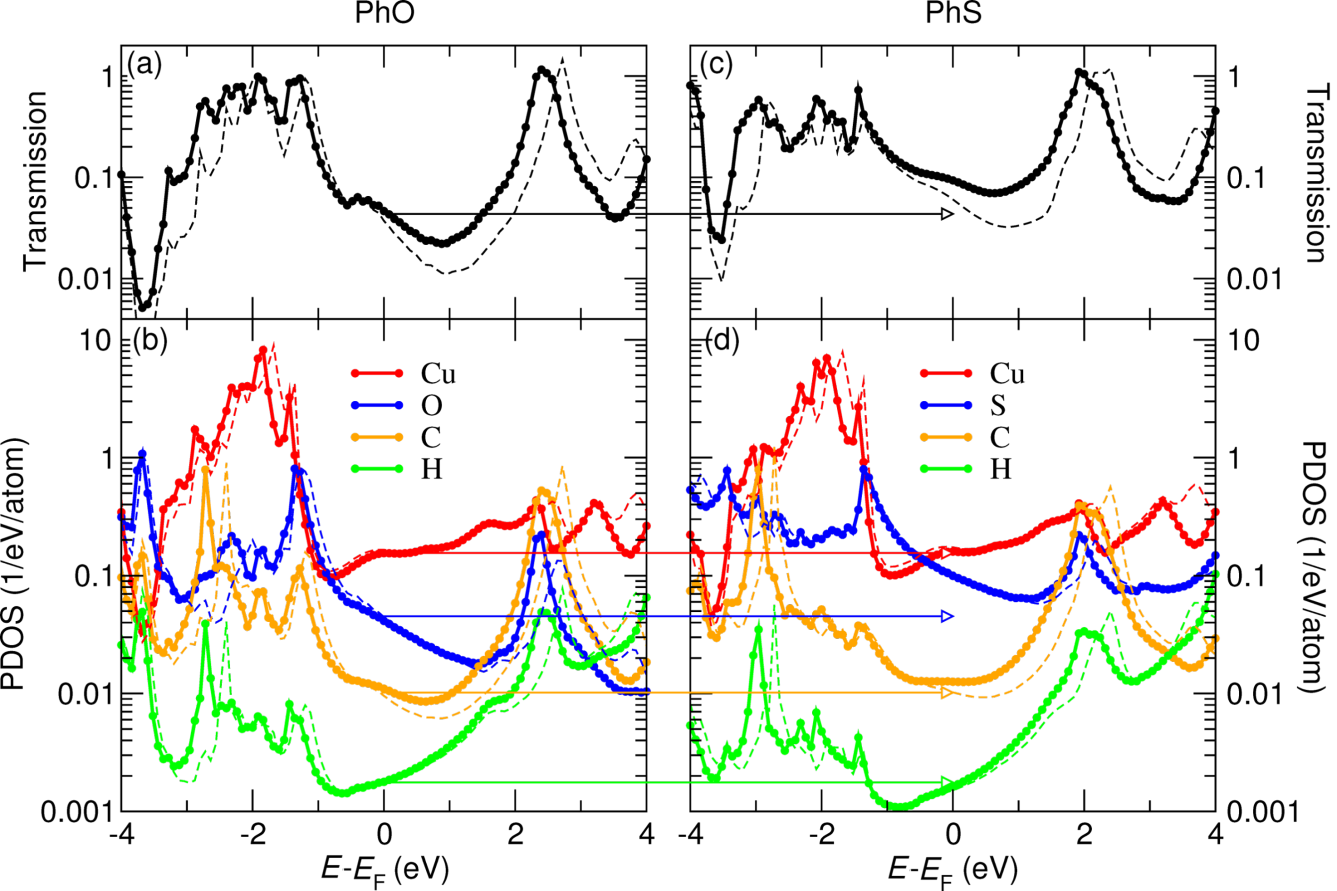
(a) Transmission and (b) projected density of states (PDOS) onto the Cu apex atom as well as onto the atoms in the PhO molecule (adapted from [12]). (c,d) Similar results for the PhS molecule. The tip height was *h* = 13.9 Å (solid lines) or *h* = 14.4 Å (dashed lines) and the lateral position was *x* = 1.4 Å. The conducting molecule is in the lifted configuration. The horizontal arrows guide the eye to the differences between PhO to PhS around *E*_F_, mainly observed in the transmission and chalcogen-site PDOS.

Our transport simulations for the PhO and PhS junctions are summarized in Figure 6. The low-bias conductance is obtained from the Landauer formula, *G* = *G*_0_*T*(*E*_F_), where *G*_0_ = 2*e*^2^/h is the conductance quantum and *T*(*E*_F_) is the transmission probability for electrons at the Fermi energy *E*_F_. Considering two different tip positions (*h* = 13.9 Å or *h* = 14.4 Å; *x* = 1.4 Å) we find conductance values of 3.7–4.7 × 10^−2^*G*_0_ (6.1–9.3 × 10^−2^*G*_0_) for the lifted PhO (PhS) molecule oriented along [001]. While the computed absolute conductances are overestimated by a factor of ≈3–4, the conductance ratio, 1.6–2.0, between the two species is in excellent agreement with the experiment. The slightly higher conductance for PhS (i.e., enhanced transmission around *E*_F_, Figure 6a,c), is ascribed to a stronger hybridization with the substrate states as revealed by the increased projected density of states (PDOS) onto the chalcogen atom near *E*_F_ (Figure 6b,d; more extended atomic orbitals of S than of O) as well as by the larger width of the lowest unoccupied molecular orbital (LUMO) resonance around 2.0–2.4 eV above *E*_F_ (Figure 6b,d).

**Figure 7:**
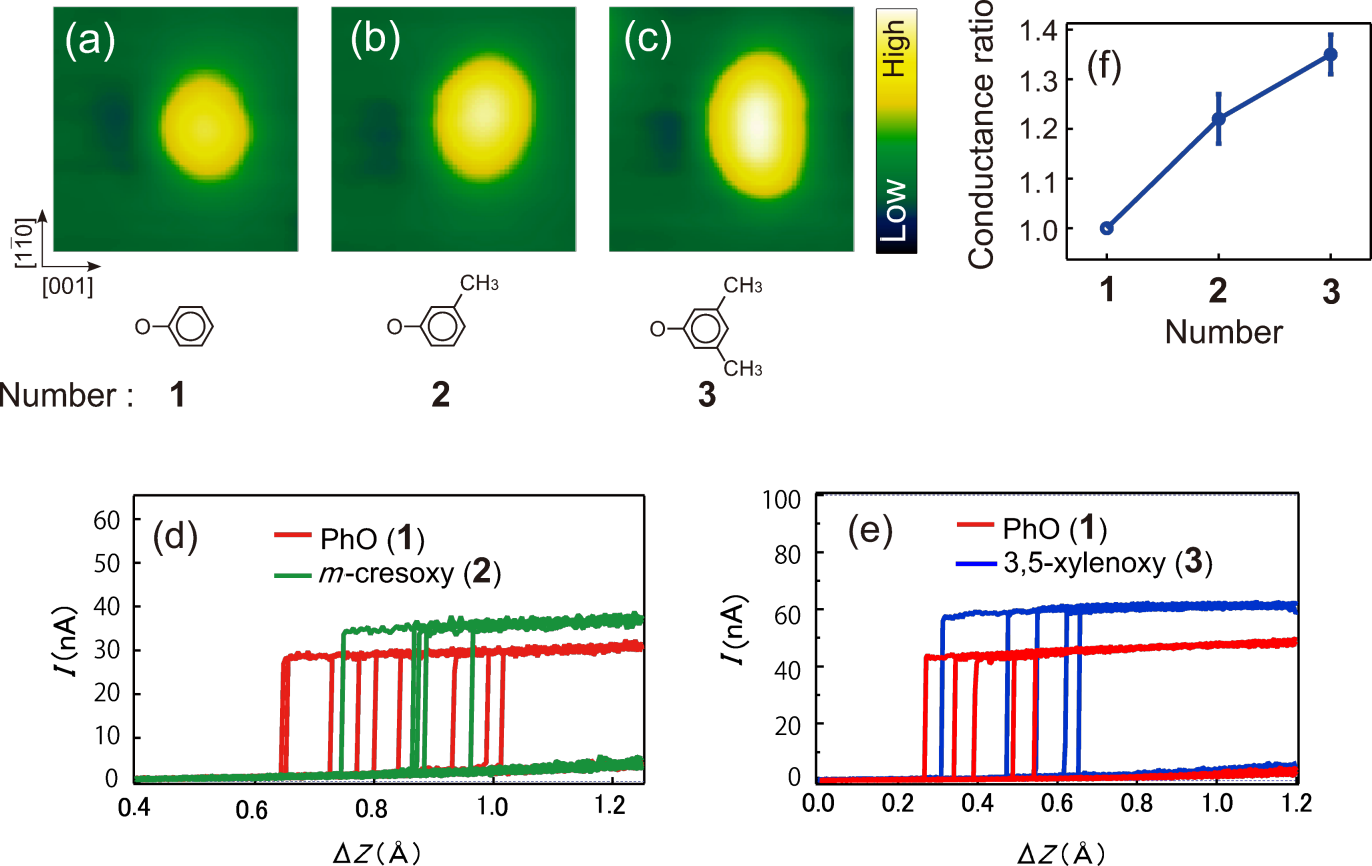
Comparison between STM images of (a) PhO, (b) *m*-cresoxy, and (c) 3,5-xylenoxy molecules on Cu(110). The images were obtained at *V*_S_ = 50 mV and *I* = 1 nA (17 × 17 Å^2^). Typical conductance plateau of (d) *m*-cresoxy and (e) 3,5-xylenoxy in comparison with that of PhO recorded with the same tip apex. The tip apexes for (d) and (e) are different and so is the conductance of PhO. (f) We compared the conductance with various tip apexes and determined the conductance ratio of *m*-cresoxy and 3,5-xylenoxy to that of PhO. The numbers 1, 2 and 3 indicate the number of molecules of PhO (unity), *m*-cresoxy and 3,5-xylenoxy, respectively.

The chemical manipulation of the on-state conductance is also feasible by introducing functional groups into the phenyl ring, which increases or decreases the energy of the electronic level of the molecule responsible for the electron conduction. While the effect of an anchoring group on the molecular conductance has been intensively studied [29–31], that of the side group to the molecular backbone has received less attention [32–34]. This is probably due to the fact that the latter is much smaller than the former. Here we explore the effect of introducing one or two methyl groups into the phenyl ring at the *meta* positions (*m*-cresoxy and 3,5-xylenoxy, respectively). The corresponding STM images are shown in Figure 7a–c along with that of PhO for comparison. The methyl group on the phenyl ring appears bright, and the molecules coexisting on the surface can therefore be discriminated by their images. Figure 7d and Figure 7e compare typical *I*–Δ*z* curves for these molecules with those obtained on PhO with an identical tip apex, clearly demonstrating the impact of the methyl groups on the conductance, which increases as much as ≈20% and ≈35% for *m*-cresoxy and 3,5-xylenoxy, respectively (Figure 7f). This effect can be understood as an electrostatic consequence of electron-donating methyl groups that push up the π orbitals, resulting in an increased conductance because of a negative slope in the transmission around *E*_F_ (Figure 6a). Thus, an increase/decrease of the on-state conductance could be rationally achieved by substituting the phenyl ring with electron donating/withdrawing functional groups [32–34].

## Conclusion

We have demonstrated a molecular switch that derives its function from the mechanical motion of a phenyl ring. The tip of a low-temperature STM was used to image and manipulate individual phenoxy (PhO) and thiophenoxy (PhS) molecules on Cu(110). These species are strongly bonded to the surface via the chalcogen atom in a nearly flat configuration. By moving the STM tip towards a target molecule or by applying a voltage pulse over it, the molecule can be lifted up to make contact to the tip apex while remaining firmly anchored to the Cu surface with the chalcogen atom acting as a hinge. Because the tip–phenyl π-bonding interaction is relatively weak compared with the covalent bond to the surface, the molecule can be released to the original position by tip retraction or by application of a reverse voltage pulse. The reversible and non-destructive operation of the switches allowed us to quantify the impact of the tip–molecule contact geometry and the chemical nature of functional groups on the molecular conductance. We observed that PhS is more conductive than PhO by a factor of 2.1, irrespective of the tip–molecule contact details. Similarly, we also studied the effect of introducing methyl groups into the phenyl ring and found that the on-state conductance could be increased by ≈35%.

The operation of these phenyl-based switches was studied by first-principles simulations, revealing the relative stabilities of the flat and lifted molecular configurations. Finite-bias calculations showed consistency with the observed tendency for the junction to prefer the on (off) state at negative (positive) sample voltages. The low-bias conductance ratio between PhO and PhS was also reproduced and ascribed to different hybridization strengths with the substrate states.

In summary, we propose that phenyl rings are promising as versatile units for molecular electronic devices, whose mechanics can be controlled by different triggers such as electric field or electrode displacement. This controlled switching can be extended to other substituted phenyl groups, allowing for rational control of the on-state conductance through chemical modification.
